# Spinal muscular atrophy type I associated with a novel *SMN1* splicing variant that disrupts the expression of the functional transcript

**DOI:** 10.3389/fneur.2023.1241195

**Published:** 2023-09-20

**Authors:** Christina Votsi, Pantelitsa Koutsou, Antonis Ververis, Anthi Georghiou, Paschalis Nicolaou, George Tanteles, Kyproula Christodoulou

**Affiliations:** ^1^Neurogenetics Department, The Cyprus Institute of Neurology and Genetics, Nicosia, Cyprus; ^2^Clinical Genetics Department, The Cyprus Institute of Neurology and Genetics, Nicosia, Cyprus

**Keywords:** novel variant, spinal muscular atrophy, Cypriot population, transcript analysis, splicing dysregulation

## Abstract

**Introduction:**

Spinal muscular atrophy (SMA) is an autosomal recessive neuromuscular disorder caused by pathogenic variants in the *SMN1* gene. The majority of SMA patients harbor a homozygous deletion of *SMN1* exon 7 (95%). Heterozygosity for a conventional variant and a deletion is rare (5%) and not easily detected, due to the highly homologous *SMN2* gene interference. *SMN2* mainly produces a truncated non-functional protein (SMN-d7) instead of the full-length functional (SMN-FL). We hereby report a novel *SMN1* splicing variant in an infant with severe SMA.

**Methods:**

MLPA was used for *SMN1/2* exon dosage determination. Sanger sequencing approaches and long-range PCR were employed to search for an *SMN1* variant. Conventional and improved Real-time PCR assays were developed for the qualitative and quantitative *SMN1/2* RNA analysis.

**Results:**

The novel *SMN1* splice-site variant c.835-8_835-5delinsG, was identified in compound heterozygosity with *SMN1* exons 7/8 deletion. RNA studies revealed complete absence of *SMN1* exon 7, thus confirming a disruptive effect of the variant on *SMN1* splicing. No expression of the functional *SMN1*-FL transcript, remarkable expression of the *SMN1-*d7 and increased levels of the *SMN2*-FL/*SMN2*-d7 transcripts were observed.

**Discussion:**

We verified the occurrence of a non-deletion *SMN1* variant and supported its pathogenicity, thus expanding the *SMN1* variants spectrum. We discuss the updated SMA genetic findings in the Cypriot population, highlighting an increased percentage of intragenic variants compared to other populations.

## Introduction

Spinal muscular atrophy (SMA) is a neuromuscular genetic disorder with autosomal recessive inheritance. It is characterized by degeneration and selective loss of lower motor neurons, thus causing muscle weakness and atrophy. It is considered one of the most common causes of infant mortality, with an estimated prevalence of approximately one to two per 100,000 individuals and an incidence of about one in 10,000 live births (1, 2). The disease is caused by pathogenic variants in the survival motor neuron gene 1 (*SMN1*) located in the telomeric position of an inverted duplication of 500 kb on chromosome 5q12.2-q13.3 (3). *SMN1* encodes the survival motor neuron (SMN) protein, a ubiquitously expressed protein involved in multiple fundamental cellular homeostatic pathways (4). A copy of this gene, known as survival motor neuron gene 2 (*SMN2*), is located in the centromeric region of this duplication. *SMN2* has high homology to *SMN1* (99% sequence identity). It differs by 16 nucleotides (5, 6), one of which (c.840C > T, exon 7, rs1164325688) results in abnormal splicing, thus producing (85–90%) a truncated delta non-functional protein (SMN-d7) (1, 5, 7). The full-length form (SMN-FL) is primarily produced by the *SMN1* gene (8). The *SMN2* gene cannot rescue the symptoms, but it is considered an important modifier of the clinical phenotype. Therefore, the disease severity is inversely correlated to the number of *SMN2* copies. Based on the age of onset, the severity of motor dysfunction and the number of *SMN2* copies, the disease has been classified into five types (0, the most severe, to 4, the mildest) (9). Furthermore, the amount of SMN-FL produced by the *SMN2* gene is modified by the presence of *SMN2* variants, which affect interactions with exonic and intronic splicing enhancers or silencers. Additional genes that act as disease modifiers by induced overexpression or downregulation have also been reported (1, 6). Different therapies targeting these modifiers have been developed for SMA patients, such as an antisense oligonucleotide (ASO) approach targeting an *SMN2* intronic splicing silencer, thereby facilitating the generation of *SMN2*-FL transcripts (1).

Most SMA patients (95%) have no *SMN1* due to a homozygous deletion encompassing exon 7 or an *SMN1* to *SMN2* conversion, which occurs more rarely. In the remaining 5% of patients, compound heterozygosity for an *SMN1* deletion and a small-scale pathogenic variant, including missense, nonsense, splice site variants, insertions, deletions and duplications, has been described (1, 9, 10). Due to the high degree of sequence homology shared by the *SMN1* and *SMN2* genes, screening for *SMN1* intragenic variants becomes more complicated, and most reported methods present limitations. Thus far, techniques such as reverse transcription (RT) PCR followed by clone sequencing (3), a long-range PCR (LR-PCR) specifically amplifying *SMN1* (7, 11) as well as an allelic specific RT-PCR (10) have been used. However, only the LR-PCR has been appropriate for detecting intronic variants and could be used upon RNA sample unavailability. Furthermore, to our knowledge, determination of the RNA transcript levels has been mainly performed by relative quantification assays using conventional or Real-Time PCR, which were not able to distinguish the *SMN1* and *SMN2* derived full-length and truncated transcripts (2, 8, 12, 13). Absolute Real-Time PCR based on TaqMan probes has also been used and efficiently determined the *SMN1* and *SMN2* full-length transcripts. However, the truncated transcripts’ levels were either not significant and, therefore, not measured (14, 15) or not distinguished (16).

We hereby describe a novel *SMN1* splice site variant that possibly affects the acceptor splice site of intron 6. The variant has been found in compound heterozygosity with *SMN1* exons 7/8 deletion, thus causing an SMA type 1 phenotype in a female patient. We also present the determination of the *SMN1* and *SMN2* full-length and truncated transcript levels by relative quantification through an improved allele-specific Real-Time PCR assay that consisted of a rapid and cost-effective way to determine the four transcript levels distinctly. Our findings support a pathogenic role for the novel variant. In addition, we discuss the up to date genetic findings of SMA in the Cypriot population and we highlight an increased percentage of intragenic variants compared to other populations.

## Materials and methods

### Molecular analyses

#### Multiplex ligation probe amplification

The Multiplex Ligation Probe Amplification (MLPA) method was used to detect the *SMN1* and *SMN2* copy numbers. The SALSA MLPA kit P021-A1 SMA (MRC-Holland, Amsterdam, Netherlands) was used according to manufacturer instructions.

#### Sanger sequencing

Primers amplifying the sequence encompassing the *SMN1/2* coding exons and intronic flanking regions were designed by us. Amplification products were sequenced in both directions using the Big Dye Terminator v1.1 Cycle Sequencing kit [Applied Biosystems (ABI), California, United States]. Sequence traces were automatically compared with the normal region sequences as listed in the GenBank database using the Seqscape software (ABI).

#### *SMN1* long-range PCR

Primers for the specific amplification of a 13.7-kb *SMN1* region, including exons 2–8, were redesigned by us based on a previous publication (11) (modifications have been performed to improve specificity, Supplementary Table S1). The GoTaq Long PCR master mix (Promega, Madison, Wisconsin, United States) was used according to manufacturer’s instructions in a 50 μL reaction volume. A touch-down PCR protocol was applied as follows: initial denaturation at 95°C for 2 min, followed by 14 cycles of denaturation at 92°C for 30 s, annealing at 63°C for 30 s, decreasing 0.5°C each cycle, extension at 68°C for 15 min, followed by 19 cycles of denaturation at 92°C for 30 s, annealing at 56°C for 30 s, extension at 68°C for 15 min and10 s, increasing 10 s per cycle, and a final extension step at 68°C for 10 min. Expected 13.7-kb products were confirmed by 0.8% agarose gel electrophoresis. Then they were excised and extracted from the gel using NucleoSpin Gel and PCR clean-up kit (MN) according to manufacturer’s instructions. The purified *SMN1*-specific product was then used as the template for Cycle Sequencing using internal primers for a short region in intron 6, encompassing the candidate variant.

#### *In-silico* prediction

Five *in-silico* prediction tools were used to predict the effect of the identified variant on splicing: Sroogle (17), ESE finder (18), Human Splicing Finder (HSF) (19), Mutation Taster (20) and NNSPLICE 0.9 (21).

#### Transcript analysis

Total RNA from the proband, a non-disease control individual, and the positive control LCLs was used for cDNA synthesis using the Protoscript First Strand cDNA Synthesis Kit [New England Biolabs (NEB), Ipswich, MA, United States]. The synthesized strands were used as substrates for transcript analysis. An initial qualitative transcript length analysis was performed by conventional PCR using primers designed on exons 6 and 8 to amplify both *SMN1* and *SMN2* transcripts, followed by agarose gel electrophoresis. PCR products also underwent digestion by the *Dde*I (NEB) endonuclease, which is often used for the *SMN1/SMN2* separation because it recognizes and cleaves the *SMN2* molecules due to the presence of a recognition site in exon 8. Agarose gel electrophoresis of the digested and undigested products enabled a comparison of the length of the transcripts, thus leading to a first estimation of the variant effect on splicing. Gel excision and extraction of the non-digested product were also performed. The purified product was sequenced using Sanger sequencing to delineate the splicing effect more accurately. Moreover, real-time PCR experiments were performed to assess the expression levels of the *SMN1*-FL*, SMN2*-FL*, SMN1*-d7*, SMN2*-d7, and the total *SMN*-d7 transcripts more accurately. The Power Track SYBR green master mix (ABI) and primer sets specific for the above transcripts (designed by us based on two of the *SMN1/SMN2* nucleotide variations, Supplementary Table S1) were used. The two housekeeping genes *B2M* and *L19* were also used as endogenous controls. Three independent real-time PCR experiments of three technical replicates for each sample were performed using the QuantStudio 7 Flex instrument (ABI). Relative expression calculations were performed using the method described by Ganger et al. (22). Statistical analysis was not performed due to sample size limitations.

#### Samples

Blood samples were obtained from the proband and her parents. Lymphoblastoid cell lines (LCLs) of an individual confirmed with a homozygous deletion of the *SMN1* gene were purchased from the Coriell Institute to be used as a positive control in the experiments. Existing internal control samples from two *SMN1* and two *SMN2* copies were also used for copy number determination and transcript analysis. DNA was extracted from samples using standard salting-out procedures. RNA was extracted from the proband and an internal control using the Nucleospin RNA blood kit [Macherey Nagel (MN), Düren, Germany]. RNA from LCLs was extracted using the Nucleospin RNA kit (MN).

## Results

### Clinical description

The proband was a 3-month-old girl born at 37^+6^ weeks gestation to first-cousin parents by C-section due to a previous section. Prior to the pregnancy with the proband, the couple had six miscarriages and a normal twin pregnancy with identical twins that enjoy good general health. There were some reduced movements antenatally compared to previous pregnancies. At birth, the patient was reported to have a weak cry with a mildly bluish skin tinge (?cyanosis). There was no significant respiratory distress reported immediately after birth but soon after the patient was intubated for the first 4 days of life. She was subsequently extubated and was started on bottle feeds. There were otherwise no reported concerns within the first 2 months; however, the paediatrician noted reduced muscle tone. The patient was subsequently reviewed by a paediatric neurologist who clinically diagnosed her with SMA. The proband then had an EMG, which was supportive of this diagnosis. At the age of 3 months, she was reported to have reduced muscle tone, and her feeding took longer. There were no concerns with bowel or bladder function. On examination, at the age of 3 months, the occipitofrontal circumference was 38.7 cm (38^th^ centile), the length was 62.5 cm (96^th^ centile), and the weight was 5 kg (19^th^ centile). The patient also had a very poor cry, mildly myopathic facies, and tongue fasciculations. Eye movements seemed normal. The palate was normal. Generalized hypotonia with a frog-leg position was also observed. She displayed very minimal movement, particularly of the left arm and none of the legs. The great toes were flexed bilaterally. Cardiovascular examination was unremarkable. The abdomen was soft and non-tender. The spine was straight. The knee deep tendon reflexes could not be elicited. The external genitalia were normal.

### Molecular analysis

A heterozygous deletion of *SMN1* exons 7 and 8 was initially detected in the proband and her father by MLPA analysis. No absence of any *SMN1* exon was detected for the mother. However, a slight decrease in the value obtained from the *SMN1* exon 7 probe hybridization was observed (0.78 instead of 1). An analogous decrease was observed for the probe on the proband (0.3 instead of 0.5), thus indicating the possible existence of a variant affecting probe hybridization efficiency. Regarding *SMN2* copy number, two copies were detected for the proband, three for the father and one for the mother.

**Figure 1:**
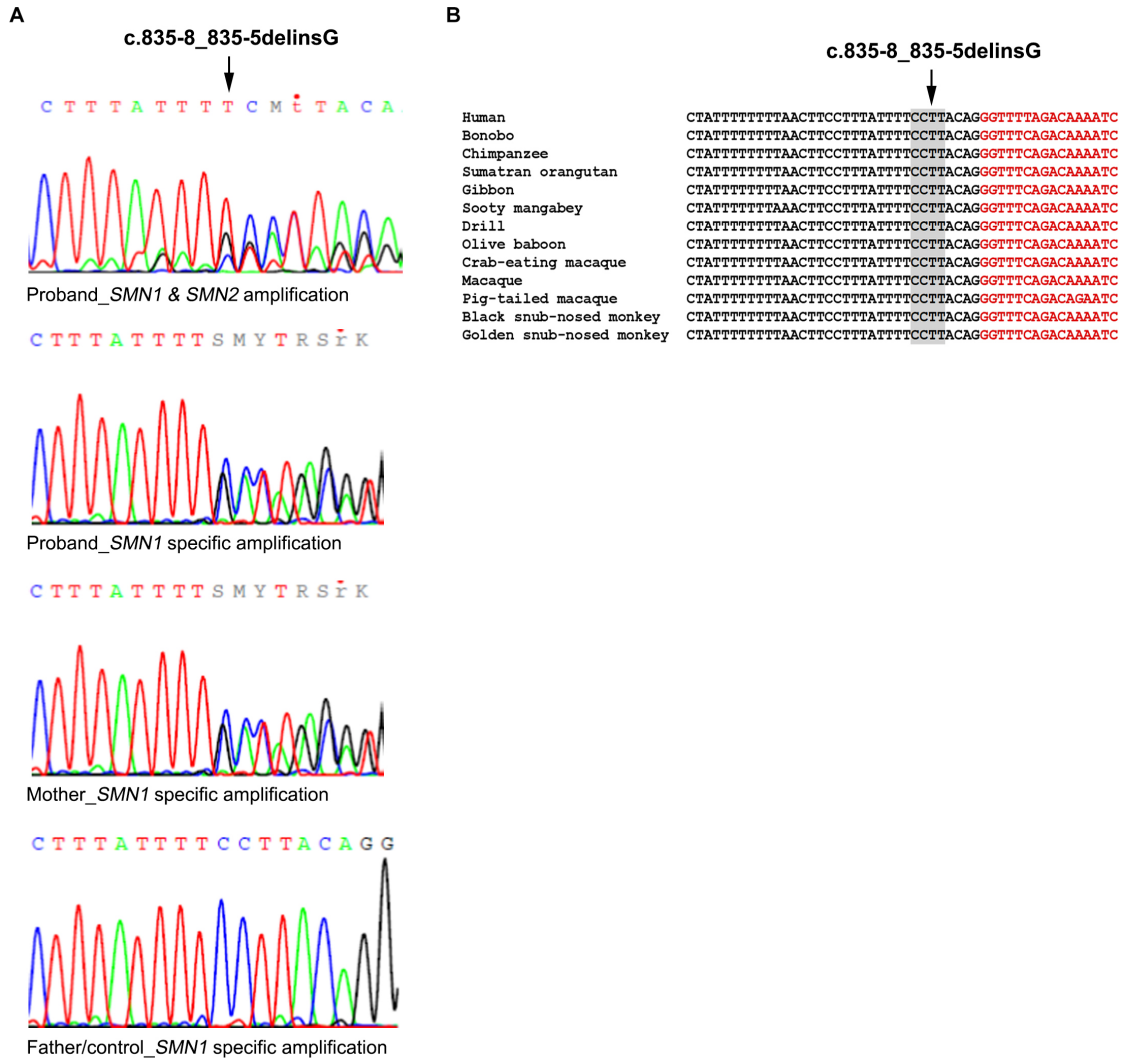
Identification of the *SMN1* novel splice site variant. **(A)** Sanger sequencing electropherograms were obtained using a non-*SMN1* specific PCR product and an *SMN1* specific LR-PCR product in the proband. The *SMN1* specific product analysis is also shown for the mother who carries the variant, the father and non-disease control representing the wild-type sequence. **(B)** Conservation of the identified variant. The DNA sequence of the *SMN1* gene region encompassing the identified variant (c.835-8_835-5delinsG) is highly conserved in various mammals. Nucleotides in black belong to intron 6 and nucleotides in red to exon 7.

Further analysis of the proband towards identifying a second intragenic variant revealed the existence of the novel *SMN1* gene splice-site NM_000344.3:c.835-8_835-5delinsG variant which has been submitted to ClinVar (accession number: SCV003935994). More specifically, the initial Sanger sequence analysis of the entire *SMN1/2* coding and flanking intronic regions in both directions excluded any small-scale variants in all coding regions. However, it was indicative of a heterozygous deletion in intron 6. A mixed sequence pattern was obtained after the deletion starting point that did not enable the accurate determination of this variant (Figure 1A). To determine if this was *SMN1* specific and also to obtain a clearer sequence pattern, LR-PCR specific for amplifying the *SMN1* gene was then performed, followed by Sanger sequencing of the region encompassing the identified variant. The LR-PCR product was gel excised and extracted and then used as the template for Sanger sequencing. Internal primers for the intron 6/exon 7 region of interest were used. A clear sequence pattern was obtained, thus enabling accurate calling of the identified novel deletion and verifying its *SMN1* specificity (Figure 1A). The same analysis performed on the parents revealed that the mother is a carrier of the deletion, whereas the father does not have this variant. Therefore, we conclude that the proband inherited the *SMN1* gene exons 7 and 8 deletion from the father and the novel c.835-8_835-5delinsG variant from the mother. This variant was absent from 200 Cypriot chromosomes and publicly available databases, including gnomAD, 1,000 Genomes, dbSNP, Ensembl, Exome Variant Server (EVS) and the Leiden Open Variation Database (LOVD). It affects a conserved splice site region (Figure 1B). *In silico* tools used to predict any effect on splicing indicated the following inconsistent predictions for the variant sequence compared to the wild type: reduced branch site, polypyrimidine track and 3′ splice site scores (Sroogle tool, Supplementary Table S2); reduced 3’splice site scores and slightly reduced score for the binding protein SRSF5 (ESE finder, Supplementary Table S3); no significant impact on splicing signals (HSF); gain of a donor site at c.835–2 position (Mutation Taster, data not shown); loss of an acceptor site at c.842 position (NNSPLICE 0.9, data not shown).

**Figure 2:**
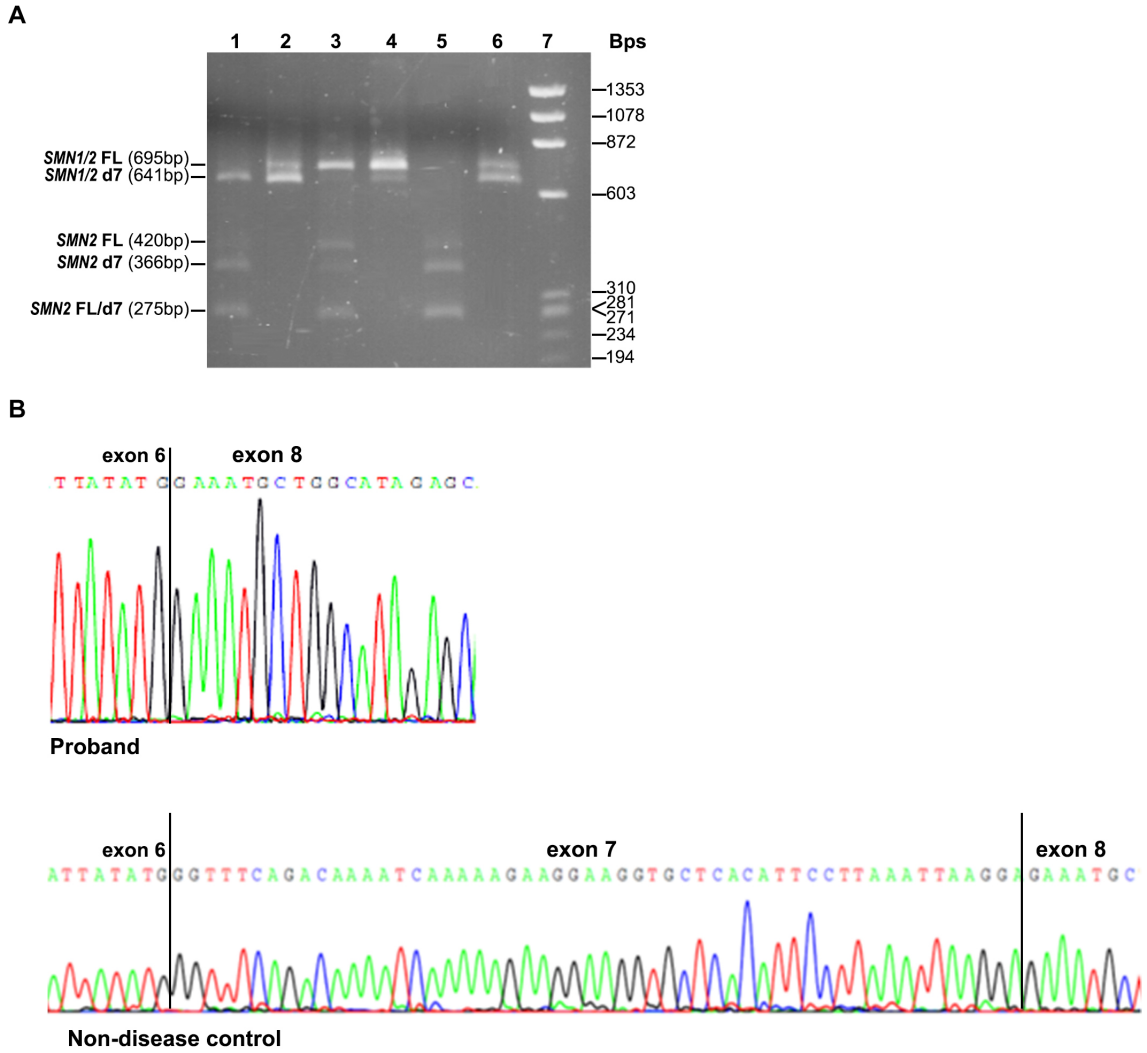
*SMN1/SMN2* transcript analysis by conventional PCR, *Dde*I digestion and Sanger sequencing. **(A)** Agarose gel electrophoresis of the undigested and *Dde*I digested PCR products that resulted from amplifying the exons 6–8 cDNA region. Digested proband = 1, Undigested proband = 2, Digested non-disease control = 3, Undigested non-disease control = 4, Digested SMA affected control = 5, Undigested SMA affected control = 6, Size standard ladder (φX174 DNA-HaeIII Digest) = 7. It is shown that a smaller band (641 bp) than the expected *SMN1/2*-FL size (695 bp) is predominant in the proband. It corresponds to the *SMN1*-d7 form as it remains undigested. In the non-disease control, the predominant is the larger size band (695 bp), which corresponds to the *SMN1*-FL form as it remains undigested. In the SMA affected control having zero *SMN1* copies, the detected bands correspond to the *SMN2*-FL and *SMN2*-d7 forms, which are fully digested (420/275 bp and 366/275 bp, respectively). **(B)** Sanger sequencing electropherograms were obtained by analyzing the remaining undigested products after performing the *Dde*I digestion in the proband and the non-disease control. The absence of exon 7 is clearly shown in the proband.

The effect of the identified variant on *SMN1* splicing and the expression of all *SMN1/SMN2* transcripts was also evaluated experimentally. Conventional PCR analysis encompassing exons 6 and 8, followed by *Dde*I digestion of the *SMN2* transcripts and agarose gel electrophoresis, indicated the absence of the *SMN1-*FL transcript and the presence of a smaller product corresponding to the *SMN1-*d7 (remaining undigested product) in the proband (Figure 2A). In contrast, in the non-disease control sample (2/2 *SMN1*/2 copies), the *SMN1*-FL had been the predominant transcript, as expected, and the *SMN1-*d7 was not easily detectable. Sanger sequencing of the gel extracted remaining undigested products (*SMN1*) in the proband and the non-disease control clearly demonstrated the absence of the entire exon 7 in the proband (Figure 2B).

**Figure 3:**
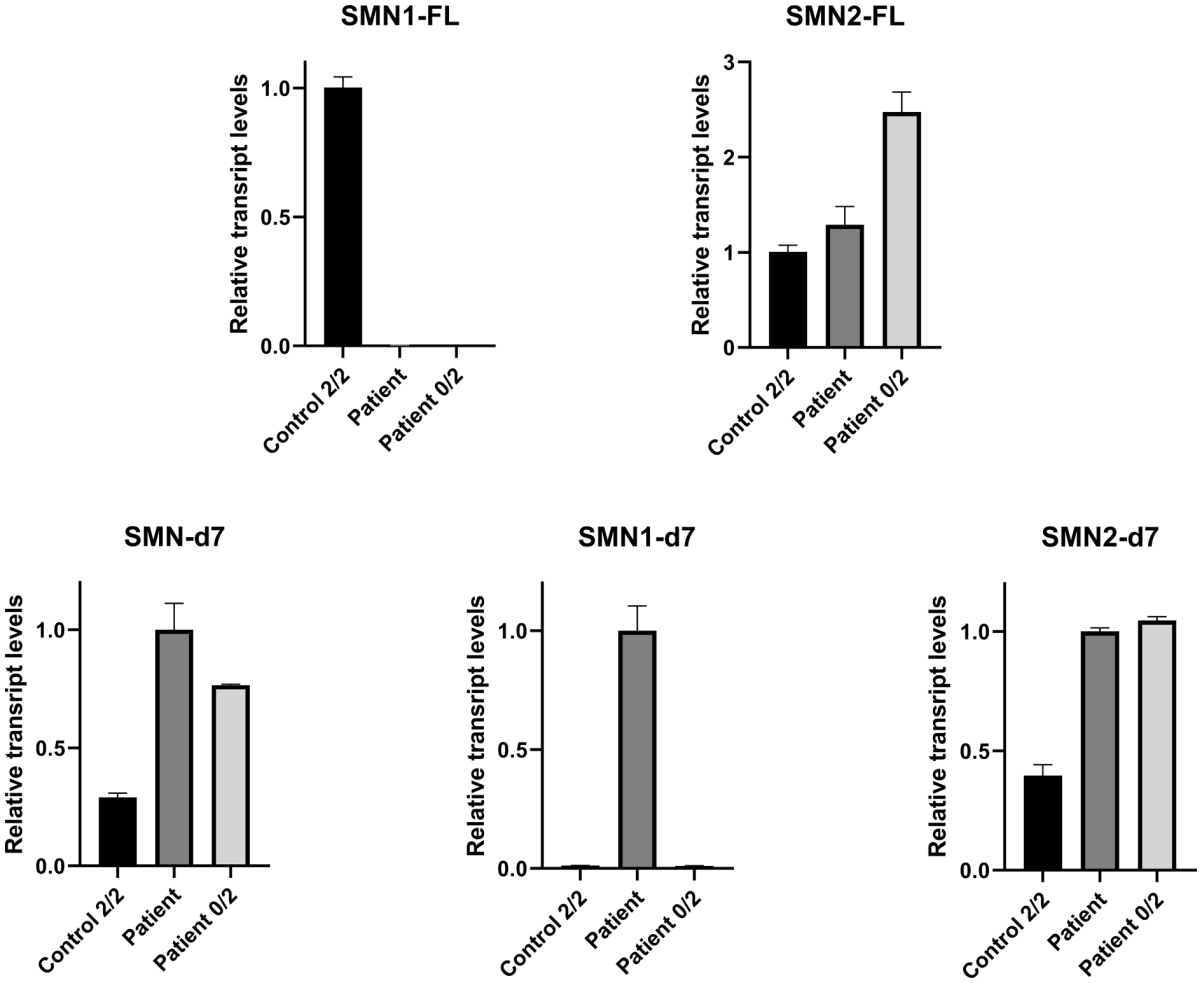
Relative mRNA expression levels of SMN transcripts in the proband (patient), the non-disease control having two copies of both *SMN1* and *SMN2* (control 2/2) and an SMA affected control having zero copies of *SMN1*, two copies of *SMN2* (patient 0/2). qPCR revealed that the functional *SMN1*-FL transcript in the proband has almost zero expression (0.003), similar to the SMA affected control (0.0002). The *SMN1*-d7 transcript was found to be expressed only in the proband, and therefore a fold-change comparison with the non-disease control was not possible. Increased levels of the *SMN2*-FL and the *SMN2*-d7 transcripts were observed for the proband (1.3 and 2.5-fold respectively) and the SMA affected control (2.5 and 2.6-fold respectively), compared to the non-disease control. Determination of the total SMN-d7 levels also agrees with the findings observed by the *SMN1*-d7 and *SMN2*-d7 distinct analyses. Increased total d7 expression was observed in the proband (3.5-fold, attributed to both the *SMN1*-d7 and *SMN2-d7* increase) and the SMA affected control (2.6-fold, attributed to the *SMN2*-d7 increase) compared to the non-disease control. Values were obtained after normalization with the *L19* and *B2M* housekeeping genes. Data are represented as the mean of three independent triplicate experiments ± SE. The non-disease control has been used as the reference sample for the interpretation of the FL relative transcripts level. For the d7 relative transcripts level interpretation, the proband has been used as the reference sample, due to the absence of the *SMN1*-d7 in the non-disease control.

Real Time-PCR experiments were designed to be specific for each one of the FL and d7 *SMN1* and *SMN2* transcripts distinctly, as well as the total *SMN*-d7. The assays were performed to evaluate the expression more accurately. This approach further demonstrated that the functional *SMN1*-FL transcript in the proband had almost zero expression, similar to the SMA-affected control sample (Figure 3). In contrast, a remarkable expression of the *SMN1-*d7 transcript was observed only in the proband. The levels of both *SMN2*-FL and *SMN2*-d7 transcripts were higher in the proband, and in the SMA affected control, compared to the non-disease control. Similar results were obtained by the determination of the total *SMN*-d7 levels (Figure 3).

## Discussion

SMA is the second most common autosomal recessive disorder and the most common genetic cause of infant mortality. It is caused by pathogenic variants in the *SMN1* gene, the most common being the homozygous deletion of exon 7 (in 95% of affected individuals). Other variants identified in compound heterozygosity with the exon 7 deletion in the majority of the remaining 5% of the patients include missense, nonsense and splice site variants, insertions, deletions and duplications (9). Due to the high degree of homology shared by *SMN1* and *SMN2*, verification of the occurrence of a non-deletion variant in the *SMN1* and not the *SMN2* is also necessary; hence, the sequencing procedure becomes more complex. Therefore, more than one approach is usually necessary to provide an accurate and early diagnosis. An early confirmed genetic diagnosis is critical since it will enable timely patient treatment.

We hereby report the identification of a novel splice site variant in compound heterozygosity with the *SMN1* exon 7 and 8 deletion in a 3-month-old girl presenting with SMA type I symptoms. During the last 30 years, a total of 16 Cypriot patients with SMA were confirmed genetically by our department which has served as the national reference centre for neurodegenerative diseases thus far. Genetic findings for the majority of them 13/16 (81.25%) have already been reported (23). Two other Cypriot cases were also reported in the past (24). In the majority (81.25%), Cypriot patients with SMA were diagnosed with an *SMN1* homozygous deletion. The currently reported variant marks the third case of intragenic variants identified in the Cypriot population. The previous two variants included a splice-site and a frameshift. The first was an intron 1 (NM_000344.3:c.81 + 1dupG) variant identified in heterozygosity with *SMN1* deletion in two siblings of a family, one affected and one unaffected. The same variant was previously detected in the Cypriot cases reported by Skordis et al. (24), and characterized as pathogenic. Therefore, phenotypic heterogeneity due to unknown modifying factors was hypothesized as a possible explanation for this family. The second variant is the exon 4 frameshift NM_000344.3:c.551_552insA, p.Lys184fsX71, which was recently identified in a family with a single affected child and was considered novel. At the same time, another group reported this variant for the first time (10) in Chinese patients. Intragenic variants usually account for 5% of SMA cases according to recent literature (1, 9, 25). With the two variants identified recently (the frameshift and the currently reported novel splice site), under the hypothesis that there are no other Cypriot cases that have been diagnosed by a foreign country centre, this percentage is ~19% in our population, which is probably much higher than in other populations To our knowledge, expect for a recent study in Brazil referring to a percentage of 10.7% of compound heterozygous intragenic variants identified by next generation sequencing without excluding the *SMN2* interference (26), the reported percentages in other populations do not exceed 5% [i.e. France (1.3%) (3), Germany (3.4%) (27), Spain (3.0–3.3%) (28, 29), Italy (3.2%) (30), Korea (3.0%) (31), United States (3.8% including possible *SMN2* variants) (32)].

The novel splice site variant was verified using a purified LR-PCR *SMN1*-specific product template for Sanger sequencing. *SMN2* interference was excluded by homozygosity of the novel variant and the NM_000344.3:c.835–44 G site of intron 6 (rs1454173648, in case of heterozygosity G/A, *SMN2* interference would exist). It is also very likely that this variant partially prevents the exon 7 MLPA probe’s regular binding thus explaining the decreased values obtained through this technique. The novel variant does not affect the dinucleotide AG of the consensus 3’acceptor splice site. However, it is near this dinucleotide, the branch site and the polypyrimidine track. Therefore, the normal binding of specific splicing proteins, which are necessary for splicing, might be affected (33). *In-silico* prediction tools did not reveal consistent changes between the variant and the wild type sequence in their majority, apart from the reduced 3′ splice site scores obtained by the Sroogle [6.43 vs. 10.92 (Max entropy), 87.09 vs. 93.34 (PSSM)] and the ESE finder tool (8.8 instead of 12.3). Therefore, we proceeded with RNA sequencing and expression experiments which finally confirmed a negative effect on mRNA splicing. RNA sequencing clearly showed exon 7 skipping. Real-Time PCR further confirmed that the truncated non-functional *SMN1-*d7 transcript was expressed instead of the functional *SMN1-*FL transcript, thus increasing the total *SMN*-d7 levels in the proband compared to the non-disease control. This finding probably results from a compensatory mechanism of the non-deleted *SMN1* allele, which fails to produce the FL transcript due to the splice site variant. Additional splice-site variants have been reported most positioned in intron 6 or intron 7 splice site regions, and in exon 7, thus causing exon 7 skipping (2, 3, 8, 12, 13, 15, 29, 34). Some of these variants directly affect the conserved donor or acceptor splice sites (2, 13, 29, 34) and some are positioned in other intronic or exonic positions different from the splice site consensus regions or the branch site. In a few cases, the consequences at the transcript level have been documented, and similar to our results, the levels of the FL transcript were reduced (2, 8, 12, 13, 15). The levels of the total d7 transcript were also reported to be increased. In most studies, the total amounts (*SMN1* and *SMN2* derived) of FL and d7 transcripts were reported, and any reductions of FL levels were attributed to *SMN1* absence. In some cases, *Dde*I digestion of the RT-PCR products followed by agarose gel electrophoresis was also performed (2, 8) to define each transcript origin, however, without providing accurate quantification. The more accurate quantification of the FL transcripts resulted from absolute relative quantification experiments based on using TaqMan probes specific for the *SMN1*-FL and *SMN2-*FL transcripts. This approach was performed either for investigating a variant effect (15) or identifying biomarkers (14, 16). However, the d7 transcripts were either not measured or not distinguished as *SMN1* or *SMN2* specific. In the current study, the four transcripts were quantified separately by relative quantification without using TaqMan probes but by using sets of primers designed based on specific nucleotide differences between *SMN1* and *SMN2*. To our knowledge, our experimental approach is novel and provides a targeted quantification.

Our findings support a pathogenic effect for the novel c.835-8_835-5delinsG splicing variant. This variant affects normal splicing and leads to exon 7 skipping, as shown by functional analysis. The critical role of exon 7 skipping in SMA development has been well demonstrated and is further supported by the current data. However, it was not possible to determine the exact mechanism of aberrant splicing. In other reported cases, a variant could lead to the disruption of an exonic splice enhancer or the generation of an exonic splicing silencer thus preventing the recruitment of splicing factors such as the SF2/ASF, U2AF and others, or promoting the binding of splicing repressors such as the hnRNP A1. It was suggested that a previously reported neighboring variant (NM_000344.3:c.835-3C > T, rs772466166) produces a shift in the splice-site similar to that of *SMN2*. The exon 8 acceptor site out-competes the one of exon 7 due to inhibitory factors encompassing the +6 variant position in exon 7 (one of the nucleotides distinct from *SMN1*) (12). Similarly, we hypothesize that the currently reported variant might suppress the exon 7 acceptor site (according to the above two consistent predictions), thus allowing the selection of the following acceptor site and the skipping of exon 7. Otherwise, the variant either affects a feature that cannot be considered by the algorithms used by the *in-silico* prediction tools or affects the RNA secondary structure, thus promoting exon 7 skipping.

In conclusion, we hereby report the identification of a novel splicing pathogenic variant in the *SMN1* gene in the Cypriot population, thus increasing the spectrum and the percentage of intragenic variants. Furthermore, the critical role of exon 7 skipping in SMA development is highlighted further through our findings. Our study provided early genetic diagnosis of the affected child, thus demonstrating the need to start treatment. The resulting data are crucial for genetic counseling, carrier testing in any first-degree family relatives and prenatal diagnosis if necessary.

## Data availability statement

The original contribution presented in the study is publicly available. This data can be found here: National Center for Biotechnology Information (NCBI) ClinVar, [https://www.ncbi.nlm.nih.gov/clinvar/, SCV003935994].

## Author contributions

CV and KC designed and conceived the study. CV carried out the experimental studies, data analysis, and interpretation. PK performed part of the experimental studies. AV, AG, and PN contributed in data analysis and interpretation. CV drafted the manuscript. GT performed the clinical characterization and drafted the relevant part. KC revised the manuscript and supervised the study. All authors read and approved the final manuscript.

## Funding

This research did not receive any specific grant from funding agencies in the public, commercial, or not-for-profit sectors. It was supported by the Cyprus Institute of Neurology and Genetics.

## Conflict of interest

The authors declare that the research was conducted in the absence of any commercial or financial relationships that could be construed as a potential conflict of interest.

## Publisher’s note

All claims expressed in this article are solely those of the authors and do not necessarily represent those of their affiliated organizations, or those of the publisher, the editors and the reviewers. Any product that may be evaluated in this article, or claim that may be made by its manufacturer, is not guaranteed or endorsed by the publisher.

## References

[1] Rouzier C Chaussenot A Paquis-Flucklinger V. Molecular diagnosis and genetic counseling for spinal muscular atrophy (SMA). Arch Pediatr. (2020) 27:7S9–7S14. doi: 10.1016/S0929-693X(20)30270-0, PMID: 33357600

[2] Ronchi D Previtali SC Sora MG Barera G Del Menico B Corti S. Novel splice-site mutation in SMN1 associated with a very severe SMA-I phenotype. J Mol Neurosci. (2015) 56:212–5. doi: 10.1007/s12031-014-0483-4, PMID: 25572663

[3] Lefebvre S Burglen L Reboullet S Clermont O Burlet P Viollet L. Identification and characterization of a spinal muscular atrophy-determining gene. Cells. (1995) 80:155–65. doi: 10.1016/0092-8674(95)90460-3, PMID: 7813012

[4] Chaytow H Huang YT Gillingwater TH Faller KME. The role of survival motor neuron protein (SMN) in protein homeostasis. Cell Mol Life Sci. (2018) 75:3877–94. doi: 10.1007/s00018-018-2849-1, PMID: 29872871 PMC6182345

[5] Butchbach MER. Genomic variability in the survival motor neuron genes (SMN1 and SMN2): implications for spinal muscular atrophy phenotype and therapeutics development. Int J Mol Sci. (2021) 22:7896. doi: 10.3390/ijms22157896, PMID: 34360669 PMC8348669

[6] Costa-Roger M Blasco-Perez L Cusco I Tizzano EF. The importance of digging into the genetics of SMN genes in the therapeutic scenario of spinal muscular atrophy. Int J Mol Sci. (2021) 22:9029. doi: 10.3390/ijms22169029, PMID: 34445733 PMC8396600

[7] Kubo Y Nishio H Saito K. A new method for SMN1 and hybrid SMN gene analysis in spinal muscular atrophy using long-range PCR followed by sequencing. J Hum Genet. (2015) 60:233–9. doi: 10.1038/jhg.2015.16, PMID: 25716911

[8] Lorson CL Hahnen E Androphy EJ Wirth B. A single nucleotide in the SMN gene regulates splicing and is responsible for spinal muscular atrophy. Proc Natl Acad Sci U S A. (1999) 96:6307–11. doi: 10.1073/pnas.96.11.6307, PMID: 10339583 PMC26877

[9] Keinath MC Prior DE Prior TW. Spinal muscular atrophy: mutations, testing, and clinical relevance. Appl Clin Genet. (2021) 14:11–25. doi: 10.2147/TACG.S239603 33531827 PMC7846873

[10] Xu Y Xiao B Liu Y Qu XX Dai MY Ying XM. Identification of novel SMN1 subtle mutations using an allelic-specific RT-PCR. Neuromuscul Disord. (2020) 30:219–26. doi: 10.1016/j.nmd.2019.11.010, PMID: 32169315

[11] Clermont O Burlet P Benit P Chanterau D Saugier-Veber P Munnich A. Molecular analysis of SMA patients without homozygous SMN1 deletions using a new strategy for identification of SMN1 subtle mutations. Hum Mutat. (2004) 24:417–27. doi: 10.1002/humu.20092, PMID: 15459957

[12] Vezain M Gerard B Drunat S Funalot B Fehrenbach S N'Guyen-Viet V. A leaky splicing mutation affecting SMN1 exon 7 inclusion explains an unexpected mild case of spinal muscular atrophy. Hum Mutat. (2011) 32:989–94. doi: 10.1002/humu.21528, PMID: 21542063

[13] Sheng-Yuan Z Xiong F Chen YJ Yan TZ Zeng J Li L. Molecular characterization of SMN copy number derived from carrier screening and from core families with SMA in a Chinese population. Eur J Hum Genet. (2010) 18:978–84. doi: 10.1038/ejhg.2010.54, PMID: 20442745 PMC2987421

[14] Tiziano FD Pinto AM Fiori S Lomastro R Messina S Bruno C. SMN transcript levels in leukocytes of SMA patients determined by absolute real-time PCR. Eur J Hum Genet. (2010) 18:52–8. doi: 10.1038/ejhg.2009.116, PMID: 19603064 PMC2987170

[15] Qu YJ Bai JL Cao YY Wang H Jin YW Du J. Mutation Spectrum of the survival of motor neuron 1 and functional analysis of variants in Chinese spinal muscular atrophy. J Mol Diagn. (2016) 18:741–52. doi: 10.1016/j.jmoldx.2016.05.004, PMID: 27425821

[16] Crawford TO Paushkin SV Kobayashi DT Forrest SJ Joyce CL Finkel RS. Evaluation of SMN protein, transcript, and copy number in the biomarkers for spinal muscular atrophy (Bfor SMA) clinical study. PLoS One. (2012) 7:e33572. doi: 10.1371/journal.pone.0033572, PMID: 22558076 PMC3338744

[17] Schwartz S Hall E Ast G. SROOGLE: webserver for integrative, user-friendly visualization of splicing signals. Nucleic Acids Res. (2009) 37:W189–92. doi: 10.1093/nar/gkp320, PMID: 19429896 PMC2703896

[18] Cartegni L Wang J Zhu Z Zhang MQ Krainer AR. ESEfinder: a web resource to identify exonic splicing enhancers. Nucleic Acids Res. (2003) 31:3568–71. doi: 10.1093/nar/gkg616, PMID: 12824367 PMC169022

[19] Desmet FO Hamroun D Lalande M Collod-Beroud G Claustres M Beroud C. Human splicing finder: an online bioinformatics tool to predict splicing signals. Nucleic Acids Res. (2009) 37:e67. doi: 10.1093/nar/gkp215, PMID: 19339519 PMC2685110

[20] Schwarz JM Cooper DN Schuelke M Seelow D. Mutation Taster2: mutation prediction for the deep-sequencing age. Nat Methods. (2014) 11:361–2. doi: 10.1038/nmeth.2890, PMID: 24681721

[21] Reese MG Eeckman FH Kulp D Haussler D. Improved splice site detection in genie. J Comput Biol. (1997) 4:311–23. doi: 10.1089/cmb.1997.4.311, PMID: 9278062

[22] Ganger MT Dietz GD Ewing SJ. A common base method for analysis of qPCR data and the application of simple blocking in qPCR experiments. BMC Bioinform. (2017) 18:534. doi: 10.1186/s12859-017-1949-5 PMC5709943 29191175

[23] Theodorou L Nicolaou P Koutsou P Georghiou A Anastasiadou V Tanteles G. Genetic findings of Cypriot spinal muscular atrophy patients. Neurol Sci. (2015) 36:1829–34. doi: 10.1007/s10072-015-2263-5, PMID: 26017350

[24] Skordis LA Dunckley MG Burglen L Campbell L Talbot K Patel S. Characterisation of novel point mutations in the survival motor neuron gene SMN, in three patients with SMA. Hum Genet. (2001) 108:356–7. doi: 10.1007/s004390100497, PMID: 11379882

[25] Nishio H Niba ETE Saito T Okamoto K Takeshima Y Awano H. Spinal muscular atrophy: the past, present, and future of diagnosis and treatment. Int J Mol Sci. (2023) 24:11939. doi: 10.3390/ijms241511939, PMID: 37569314 PMC10418635

[26] Mendonca RH Matsui C Jr Polido GJ Silva AMS Kulikowski L Torchio Dias A. Intragenic variants in the SMN1 gene determine the clinical phenotype in 5q spinal muscular atrophy. Neurol Genet. (2020) 6:e505. doi: 10.1212/NXG.0000000000000505, PMID: 33062891 PMC7524579

[27] Wirth B Herz M Wetter A Moskau S Hahnen E Rudnik-Schoneborn S. Quantitative analysis of survival motor neuron copies: identification of subtle SMN1 mutations in patients with spinal muscular atrophy, genotype-phenotype correlation, and implications for genetic counseling. Am J Hum Genet. (1999) 64:1340–56. doi: 10.1086/302369, PMID: 10205265 PMC1377870

[28] Cusco I Lopez E Soler-Botija C Jesus Barcelo M Baiget M Tizzano EF. A genetic and phenotypic analysis in Spanish spinal muscular atrophy patients with c.399_402del AGAG, the most frequently found subtle mutation in the SMN1 gene. Hum Mutat. (2003) 22:136–43. doi: 10.1002/humu.10245 12872254

[29] Martin Y Valero A del Castillo E Pascual SI Hernandez-Chico C. Genetic study of SMA patients without homozygous SMN1 deletions: identification of compound heterozygotes and characterisation of novel intragenic SMN1 mutations. Hum Genet. (2002) 110:257–63. doi: 10.1007/s00439-002-0681-y, PMID: 11935338

[30] Brahe C Clermont O Zappata S Tiziano F Melki J Neri G. Frameshift mutation in the survival motor neuron gene in a severe case of SMA type I. Hum Mol Genet. (1996) 5:1971–6. doi: 10.1093/hmg/5.12.1971, PMID: 8968751

[31] Ahn EJ Yum MS Kim EH Yoo HW Lee BH Kim GH. Genotype-phenotype correlation of SMN1 and NAIP deletions in Korean patients with spinal muscular atrophy. J Clin Neurol. (2017) 13:27–31. doi: 10.3988/jcn.2017.13.1.27, PMID: 27730768 PMC5242148

[32] Bowen BM Truty R Aradhya S Bristow SL Johnson BA Morales A. SMA identified: clinical and molecular findings from a sponsored testing program for spinal muscular atrophy in more than 2,000 individuals. Front Neurol. (2021) 12:663911. doi: 10.3389/fneur.2021.663911, PMID: 34025568 PMC8134668

[33] Abramowicz A Gos M. Splicing mutations in human genetic disorders: examples, detection, and confirmation. J Appl Genet. (2018) 59:253–68. doi: 10.1007/s13353-018-0444-7, PMID: 29680930 PMC6060985

[34] Eggermann T Eggermann K Elbracht M Zerres K Rudnik-Schoneborn S. A new splice site mutation in the SMN1 gene causes discrepant results in SMN1 deletion screening approaches. Neuromuscul Disord. (2008) 18:146–9. doi: 10.1016/j.nmd.2007.10.003, PMID: 18155522

