# Supplementary material for: Spinal muscular atrophy type I associated with a novel SMN1 splicing variant that disrupts the expression of the functional transcript
Source: Front Neurol. 2023 Sep 20;14:1241195. doi: 10.3389/fneur.2023.1241195 (PMC10548546; doi:10.3389/fneur.2023.1241195)
Supplement: Supplementary file 1 [file Table_1.DOCX]

Supplementary Material

Spinal muscular atrophy type I associated with a novel *SMN1* splicing variant that disrupts the expression of the functional transcript

Christina Votsi, Pantelitsa Koutsou, Antonis Ververis, Anthi Georghiou, Paschalis Nicolaou, George Tanteles, Kyproula Christodoulou^*^

*** Correspondence:** Kyproula Christodoulou: [roula@cing.ac.cy](mailto:roula@cing.ac.cy)

**Supplementary Table S1:** Primers sequences used in the study

| Target for amplification | Primer | Sequence 5’ – 3’ |
| --- | --- | --- |
| *SMN1* exon 2-8 | Fw: SMN1-2ext2F | TAGGGGCATTCACTTGATGG |
|  | Rv: SMN7tel.Rbmod | CCTTCCTTCTTTTTGATTTTGGCTG |
| *SMN1-2 cDNA ex 6-8* | Fw: SMNcDNA_ex6-8_F | TCCATTTCCTTCTGGACCAC |
|  | Rv: SMNcDNA_ex6-8_R | CAATGAACAGCCATGTCCAC |
| *SMN1*-FL transcript | Fw: SMN1cDNAFL_Fmod | ATACTGGCTATTATATGGGTCTC |
|  | Rv: SMN1cDNAFL_Rmod | TGCTGGCCTCCCACCCCCATCC |
| *SMN2*-FL transcript | Fw: SMN2cDNAFL_Fmod | ATACTGGCTATTATATGGGTCTT |
|  | Rv: SMN2cDNAFL_Rmod | TGCTGGCCTCCCACCCCCAACT |
| *SMN1*-d7 transcript | Fw: SMNcDNAd7_F | TACTGGCTATTATATGGAAATGC |
|  | Rv: SMN1cDNAFL_Rmod | TGCTGGCCTCCCACCCCCATCC |
| *SMN2*-d7 transcript | Fw: SMNcDNAd7_F | TACTGGCTATTATATGGAAATGC |
|  | Rv: SMN2cDNAFL_Rmod | TGCTGGCCTCCCACCCCCAACT |
| Total *SMN*-d7 transcript | Fw: SMNcDNAd7_F | TACTGGCTATTATATGGAAATGC |
|  | Rv: SMNcDNAd7_R | AGAGTTACCCATTCCACTTCCT |

**Supplementary table S2:** Summary of the branch site, the polypyrimidine tract, the 3’ and 5’ splice sites scores obtained by the use of the Sroogle prediction tool.

| Element | *Start | End | Score | (method used) | Score Percentile (Const Exons) | Score Percentile (Alt Exons) |
| --- | --- | --- | --- | --- | --- | --- |
| Branch site | 5738 | 5744 | 3.45 | (Kol et al) | 0.8 | 0.82 |
|  | 5714 | 5720 | **2.35** | (Kol et al) | 0.02 | 0.03 |
| Polypyrimidine tract | 5745 | 5761 | 0.94 | (Kol et al) | 0.95 | 0.94 |
|  | 5722 | 5757 | **0.77** | (Kol et al) | 0.39 | 0.42 |
|  | 5731 | 5760 | 16.13 | (Schwartz et al) | 0.9 | 0.85 |
|  | 5731 | 5756 | **12.46** | (Schwartz et al) | 0.71 | 0.71 |
| 3' splice site | 5745 | 5767 | 10.92 | (Max entropy) | 0.83 | 0.85 |
|  |  |  | 93.34 | (PSSM) | 0.99 | 0.98 |
|  | 5742 | 5764 | **6.43** | (Max entropy) | 0.17 | 0.25 |
|  |  |  | **87.09** | (PSSM) | 0.62 | 0.68 |
| 5' splice site | 5816 | 5824 | -5.30 | (Delta-G) | 0.41 | 0.45 |
|  |  |  | 8.57 | (Max entropy) | 0.45 | 0.53 |
|  |  |  | 75.78 | (Senepathy) | 0.2 | 0.24 |
|  | 5813  variant sequence  Wild-type sequence | 5821 | -5.30 | (Delta-G) | 0.41 | 0.45 |
|  |  |  | 8.57 | (Max entropy) | 0.45 | 0.53 |
|  |  |  | 75.78 | (Senepathy) | 0.2 | 0.24 |

*Coordinate numbering according to intron 6 bp number (starting position= 1^st^ bp of intron 6)

| Motif | Site | Score |
| --- | --- | --- |
| 5SS_U2_human | gccagcattatgaaagtgaatcttactttt | 5.47960 |
|  | gccagcattatgaaagtgaatcttactttt | 5.47960 |
| 3SS_U2_human | ttattttccttacagGGTTTCAGACAAAAT | 12.31600 |
|  | cctttattttgacagGGTTTCAGACAAAAT | **8.87780** |
| BranchSite | TGCTCAC | 5.84930 |
|  | TGCTCAC | 5.84930 |
|  | | |
| SRSF1 | CAGACAA | 3.76512 |
|  | CAGACAA | 3.76512 |
| SRSF1 (IgM-BRCA1) | CAGACAA | 3.23999 |
|  | CAGACAA | 3.23999 |
| SRSF2 | agccactg | 4.22717 |
|  | agccactg | 4.22717 |
| SRSF5 | ttacagG | 6.01235 |
|  | tgacagG | **5.50286** |
| SRSF6 | tgcagc | 4.71335 |
|  | tgcagc | 4.71335 |

**Supplementary table S3:** Summary of the 5’ and 3’ splice site, the branch site, as well as the binding proteins SRSF1, SRSF1 (IgM-BRCA1), SRSF2, SRSF5 and SRSF6 scores, obtained by the use of the ESE finder prediction tool.

Wild-type sequence

variant sequence
